# Are doctors protected enough during COVID-19 in South Asia?

**DOI:** 10.1186/s41256-021-00219-x

**Published:** 2021-09-30

**Authors:** Nadia Nazir Jatoi, Saniya Ahmad, Emad ud-din Sajid, Farah Yasmin, Muhammad Sohaib Asghar, Syed Ali Farhan, Bushra Zafar Sayeed, Momina Mariam Marufi, Kaneez Fatima, Syed Faisal Mahmood

**Affiliations:** 1 Department of Internal Medicine, Doctor Ruth K.M. Pfau Civil Hospital, Dow University of Health Sciences, Baba-e-Urdu Road, Karachi, Sindh 74200 Pakistan; 2 Department of Internal Medicine, Dow Ojha University Hospital, Suparco Road, KDA Scheme 33, Karachi, Sindh 75300 Pakistan; 3 Department of Surgery, Dow University of Health Sciences, Baba-e-Urdu Road, Karachi, Sindh 74200 Pakistan; 4 Division of Infectious Disease, Department of Medicine, Agha Khan University Hospital, National Stadium Road, Karachi, Sindh 74800 Pakistan

## Abstract

**Background:**

The highly contagious nature of the severe acute respiratory syndrome coronavirus 2 (SARS-CoV-2) places physicians in South Asia at high risk of contracting the infection. Accordingly, we conducted this study to provide an updated account of physician deaths in South Asia during the COVID-19 pandemic and to analyze and compare the different characteristics associated with physician mortality amongst the countries of the region.

**Methods:**

We performed a cross-sectional study by using published news reports on the websites of news agencies from 9 selected countries in South Asia. Our study included only those physicians and doctors who died after contracting COVID-19 from their respective workplaces. All available data about the country of origin, type of, sex, age, medical or surgical specialty, and date of death were included.

**Results:**

The total number of physician deaths reported due to COVID-19 in our study was 170, with half (87/170, 51%) of the deaths reported from Iran. Male physician deaths were reported to be 145 (145/170 = 85%). Internal Medicine (58.43%) was the most severely affected sub-specialty. The highest physician mortality rate in the general population recorded in Afghanistan (27/1000 deaths). General physicians from India [OR = 11.00(95% CI = 1.06–114.08), *p* = 0.045] and public sector medical practitioners from Pakistan [aOR = 4.52 (95% CI = 1.18–17.33), *p* = 0.028] were showing significant mortality when compared with other regions in multivariate logistic regression.

**Conclusion:**

An increased number of physician deaths, owing to COVID-19, has been shown in South Asia. This could be due to decreased personal protective equipment and the poor health care management systems of the countries in the region to combat the pandemic. Future studies should provide detailed information of characteristics associated with physician mortalities along with the main complications arising due to the virus.

## Background

According to 2018 statistics, the Physician Density (per 10,000) of the respective countries in South Asia is 12 medical doctors per 10,000 in Pakistan, 6 medical doctors per 10,000 in Bangladesh, 9 medical doctors per 10,000 in India, 11 medical doctors per 10,000 in Sri Lanka, 16 medical doctors per 10,000 in Iran and 17 medical doctors per 10,000 in the Maldives [1].

Doctors in South Asia face innumerable challenges. Bangladesh, India, Nepal, Pakistan, and Sri Lanka, which constitute a quarter of the world’s population, have an insufficient investment in facilities and low precedence for specialty [3]. Moreover, low life expectancy, high rates of malnourishment coupled with infant mortality, the incidence of Tuberculosis and Human Immunodeficiency Virus / Acquired Immunodeficiency Syndrome (HIV/AIDS), poor sanitation, maternal health, and access to healthcare services, pose a significant health challenge [4]. Resource constraints are one of the important barriers to quality healthcare in addition to mal-governance, negligence, and unaccountability [2].

The South Asian region faces a tough battle in the face of the COVID-19 pandemic and it is indeed hardest for regions like Africa and South Asia to prevent further deterioration of the healthcare system caused by the virus [5]. The highly contagious nature of the virus places physicians on the frontline at high risk of contracting the infection. Evidently, during outbreaks of infectious diseases, the Health Care Community faces the maximum disadvantage [6]. As airway interventions for many COVID-19 patients generate aerosol, physicians are particularly at increased risk of contracting the illness [7]. Moreover, Personal Protective Equipment (PPE) at all times is highly necessary and its scarcity threatens the safety of physicians working in close contact with infected patients or other co-workers [7].

The doctor who first warned the public of SARS-like disease died of the later confirmed COVID-19 after being infected when he had treated a unkown pueumonia infected patient in Wuhan, China [8]. Thereafter, many physician fatalities were reported from China and other countries affected by the pandemic [9]. This raises the question of whether and to what extent occupational exposure to COVID-19 contributes to physicians’ mortality [10]. Investigations of risks associated with occupational health makes an integral component of occupational safety and health management [11]. Accordingly, we conducted this research keeping the following aims and objectives in our mind: (1) to provide an updated and detailed account of the most important characteristic of our study i.e. physician deaths in South Asia during the COVID-19 pandemic; (2) to analyze and compare the different characteristics associated with physician mortality amongst the different countries of the region.

## Methods

### Study design

Our study design is a cross-sectional study and therefore, the Strengthening the Reporting of Observational Studies in Epidemiology (STROBE) guidelines were utilized to construct our manuscript [12]. An Institutional Review Board approval was not needed for our review study since the evaluated data were publicly available.

### Inclusion and exclusion criteria

Our study included only those physicians who died after contracting COVID-19 from their respective workplaces. The level of graduate medical education was not a criterion of inclusion in our study and thus we included reports of deaths ranging from Medical Interns to Consultant Physicians. In some countries of South Asia, the term ‘Medical Intern’ is used interchangeably with a similar term i.e. ‘House Officer’. However, for the sake of simplicity and reporting purposes, we used the term ‘Medical Intern’ in our study. Retired physicians and doctors who were working remotely from their homes and thereafter, died from COVID-19 during the current pandemic were excluded. The words ‘doctor’ and ‘physician’ were used interchangeably in our study.

### Study variables

All available data about the country of origin, type of practice (private or public practice), sex, age, specialty (based on the description in the original news outlet source), and date of death were included. The public practice was defined as hospitals that were functioning under government or military administration, whereas private practice was defined as hospitals or clinics that were functioning autonomously. If the physician's name was reported but the sex was not mentioned, then attempts were made to identify the gender using an online tool [13]. Duplicated reports were aggregated by cross-checking the names of medical doctors. For analytical purposes, we calculated the total ‘mortality ratio of physicians’ of South Asia, as well as of individual countries by utilizing the following formula:

$$Mortality \,Ratio\, = \,\frac{{\left( {Total\,COVID\, - \,19\,deaths\,in\,physicians} \right)}}{{\left( {Total\,COVID\, - \,19\,deaths\,in\,the\,general\,population} \right)}} \times 1000$$

[14].

### Data collection

We collected the data concerning the death of doctors and physicians due to COVID-19 from published news reports on their respective newspaper agencies' websites. The collected data in our study were accurate as of August 31st, 2020. Data regarding COVID-19 deaths in the general population of each respective country were extracted from the WHO COVID-19 dashboard [14]. The methodology of data collection concerning physician mortality has been previously validated [11, 15].

We selected the following 9 countries in Southern Asia regions based on the classification of the United Nations statistics division [16]: Afghanistan, Bangladesh, Bhutan, India, Iran, Maldives, Nepal, Pakistan, and Sri Lanka. Our Google search included keywords, ‘Physician’, ‘doctor’, ‘COVID-19’, ‘Coronavirus’, ‘die’, ‘death’ in English, and was performed on June 12, 2020. The first 100 web pages, excluding the advertisement area, were used for further evaluation. Also, different news reports that were published in local languages of the respective countries were searched by the same keywords and translated using ‘Google Translate’. Official institutional websites or social media portals (i.e., LinkedIn, ResearchGate, and Twitter) were also cross-checked by writing the doctor’s name to ensure no data were missed.

Two independent investigators (E.S and S.A.) extracted data and their lists were compared to ensure accuracy of data. A third investigator (S.A.F) was consulted to resolve any inconsistency.

### Statistical analysis

The extracted data were arranged on a Microsoft Excel spreadsheet (Version 15.37, Microsoft Corporation, Redmond, WA) and analyzed by utilizing SPSS version 23 (IBM SPSS Statistics, IBM Corporation, Armonk, New York). Descriptive variables were reported as frequency and percentage. Median age of death was reported and one-way ANOVA was applied to calculate *p*-value. Since data were found non-normally distributed according to Kolmogorov–Smirnov and Shapiro–Wilk test, non-parametric distribution was also presented through Kruskal–Wallis H test. P-value of ≤ 0.05 was considered to be significant. Multivariate logistic regression model of study variables based on country of origin were conducted with adjustment to age and gender. Model 1 is univariate regression obtaining crude odds ratio (95% confidence interval) for variables, while Model 2 is multivariate regression that is adjusted for age by obtaining adjusted odds ratio (95% confidence ratio), and Model 3 is adjusted for both age and gender in multivariate logistic regression reporting adjusted odds ratio (95% confidence interval).

## Results

### Study characteristics

The total number of physician deaths reported due to COVID-19 was 170 and the reported physician deaths in Sri Lanka, Bhutan, Nepal, and the Maldives were none. The date of reported deaths in our study ranged from 6th March 2020 to 21st July 2020.

**Fig. 1:**
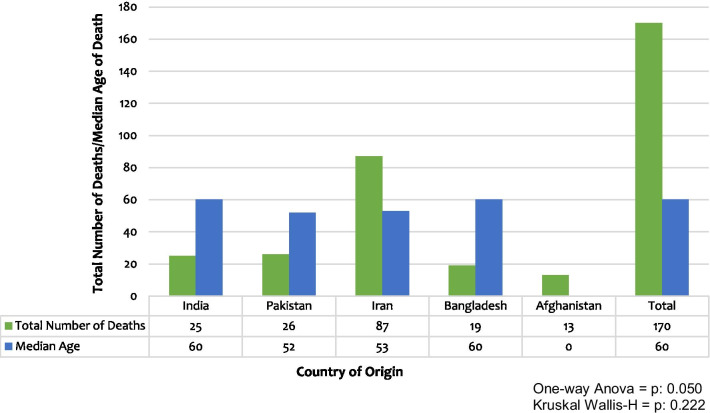
Relationship between country of origin with total number of deaths and median age of death. Bars illustrate the total number of deaths and median age at the time of death of all physicians according to their country of origin as well as a cumulative total of all countries

The number of men reported dead was 145 (85%). Sex information was not available for 13 (8%) physicians. The median age of physicians in our study was 60 years (Inter-Quartile Range = 14). The median age of men was 60 years, whereas that of women was 53 years. The age information of 121 physicians was not available. Half of the deaths reported in our study were from Iran (87/170 = 51%), followed by Pakistan (26/170 = 15%) and India (25/170 = 15%) as shown in Fig. 1. The information about the physician specialty of 35 physicians was not available. The physician specialties with the most number of physician deaths were Internal Medicine (58/135 = 43%), Pediatrics (12/135 = 9%), and Anesthesiology (11/135 = 8%). Proportionally, every seven out of ten doctors (99/135 = 73%) died due to COVID-19, belonged to an Internal Medicine specialty.

### Country-based analysis of study variables

The country with the lowest median age of died physicians was Pakistan (52  years), closely followed by Iran (53 years) as shown in Table 1. Iran recorded the highest number (81) and proportion (56%) of deaths amongst men. 4 out of the 5 deaths of Medical Interns were reported from Pakistan. More than half (59%) of the general physician deaths were reported from Iran. The majority of the deaths in India (72%) and Bangladesh (58%) were reported in physicians who practiced in a private setup. However, the majority of physicians in Pakistan (69%) were affiliated with public-sector hospitals. In our study, the mean mortality rate of physicians was calculated to be 7 physician deaths per 1000 deaths among the general population. Country-wise, the highest physician mortality rate per 1000 deaths in the general population was recorded in Afghanistan (27), followed by Bangladesh (16) and Iran (10).Table 1 breaks down these study variables based on the available data from countries of South Asia.

**Table 1:** Breakdown of study variables based on country of origin

Study variables	India n = 25 (15%)	Pakistan n = 26 (15%)	Iran n = 87 (51%)	Bangladesh n = 19 (11%)	Afghanistan n = 13 (8%)	Total n = 170 (100%)
*Gender*						
Males	22 (88%)	22 (85%)	81(93%)	16(84%)	4(31%)	145(85%)
Females	0 (0%)	4(15%)	6(7%)	2(11%)	0(0%)	12(7%)
N/A	3(12%)	0(0%)	0(0%)	1(5%)	9(69%)	13(8%)
*Physician specialty*						
Anesthesiologist	0(0%)	3(11.5%)	5(6%)	3(16%)	0(0%)	11(6.5%)
Cardiologist	0 (0%)	1(4%)	0(0%)	0(0%)	0(0%)	1(0.5%)
Pulmonary and critical care specialist	1(4%)	2(7.5%)	5(6%)	1(5%)	0(0%)	9(5.5%)
Dermatologist	0(0%)	1(4%)	1(1%)	0(%)	0(%)	2(1%)
Otolaryngologists	0(0%)	1(4%)	2(2.5%)	0(0%)	0(0%)	3(2%)
General Physician	11(44%)	8(31%)	34(39%)	3(16%)	2(15%)	58(34%)
Gynecologist/obstetrician	0(0%)	1(4%)	2(2.5%)	2(11%)	0(0%)	5(3%)
Hematologist/oncologist	0(0%)	0(0%)	2(2.5%)	0(0%)	0(0%)	2(1%)
Infectious disease specialist	0(0%)	0(0%)	1(1%)	0(0%)	0(0%)	1(0.5%)
Medical intern	0(0%)	4(15%)	0(0%)	1(5%)	0(0%)	5(3%)
Neurologist	1(4%)	0(0%)	0(0%)	0(0%)	0(0%)	1(0.5%)
Ophthalmologist	0(0%)	0(0%)	2(2.5%)	0(0%)	0(0%)	2(1%)
Orthopedic surgeon	3(12%)	0(0%)	1(1%)	2(11%)	0(0%)	6(3.5%)
Pediatrician	0(0%)	2(7.5%)	8(9%)	1(5%)	1(8%)	12(7%)
Pathologist	0(0%)	2(7.5%)	1(1%)	1(5%)	0(0%)	4(2%)
Psychiatrist	0(0%)	0(0%)	3(3%)	0(0%)	0(0%)	3(2%)
Radiologist	0(0%)	0(0%)	0(0%)	1(5%)	0(0%)	1(0.5%)
General surgeon	0(0%)	1(4%)	7(8%)	0(0%)	1(8%)	9(5.5%)
N/A	9(36%)	0(0%)	13(15%)	4(21%)	9(69%)	35(21%)
*Type of practice*						
Public	3(12%)	18(69%)	4(5%)	5(26%)	3(23%)	33(19%)
Private	18(72%)	7(27%)	14(16%)	11(58%)	1(8%)	51(30%)
N/A	4(16%)	1(4%)	69(79%)	3(16%)	9(69%)	86(51%)
Mortality rate						
Per 1000 deaths	2.5	9	10	16	27	7

### Univariate and multivariate logistic regression

On univariate analysis, male gender was found associated with higher mortality in Iran [OR = 8.10 (95% CI = 2.70–24.22), *p* < 0.001] and India [OR = 4.40 (95% CI = 1.08–17.88), *p* = 0.038]. Similarly, Public sector doctors from Pakistan [OR = 3.58 (95% CI = 1.10–13.46), *p* = 0.034] and general physicians from India [OR = 11.00 (95% CI = 1.06–114.08), *p* = 0.045] were also found associated with higher mortality as shown in Table 2. When adjusted for age, public sector doctors from Pakistan were still significantly associated with mortality [OR = 4.24 (95% CI = 1.13–15.85), *p* = 0.031], and further adjusted for age and gender [OR = 4.52 (95% CI = 1.18–17.33), *p* = 0.028]. While general physicians from India lost their significant association with mortality when adjusted for age and gender. The rest of all variables were found insignificantly associated with mortality. Values in bold in Table 2 were deemed to be statistically significant (p < 0.05).

**Table 2:** Multivariate logistic regression model of study variables based on country of origin with adjustment to age and gender

Variables	Pakistan (n = 25)			India (n = 26)			Iran (n = 87)		
	Model 1	Model 2	Model 3	Model 1	Model 2	Model 3	Model 1	Model 2	Model 3
*Gender*									
Female	1.000	–	–	1.000	–	–	1.000	–	–
Male	3.30(0.91–11.91)	–	–	4.40(1.08–17.88)	–	–	8.10(2.70–24.22)	–	–
*p*-value	0.068	–	–	**0.038**	–	–	** < 0.001**	–	–
*Type of practice*									
Private	1.000	1.000	1.000	1.000	1.000	1.000	1.000	1.000	1.000
Public	3.58(1.10–13.46)	4.24(1.13–15.85)	4.52(1.18–17.33)	0.25(0.05–1.13)	0.30(0.05–26.74)	0.31(0.06–1.63)	0.43(0.10–1.78)	0.40(0.09–1.80)	0.41(0.09–1.86)
*p*-value	**0.034**	**0.031**	**0.028**	0.073	0.155	0.171	0.244	0.236	0.252
*Specialty*									
Surgeons^a^	0.25(0.02–3.04)	0.17(0.13–2.29)	0.20(0.15–2.79)	6.00(0.42–85.24)	5.74(0.37–87.16)	4.60(0.30–70.49)	0.76(0.15–3.80)	1.86(0.24–14.21)	2.13(0.26–17.44)
*p*-value	0.277	0.183	0.234	0.186	0.208	0.273	0.740	0.548	0.478
Physicians^b^	1.50(0.35–6.34)	0.99(0.20–4.80)	1.06(0.21–5.14)	11.00(1.06–114.08)	10.03(0.91–109.52)	8.38(0.76–91.37)	1.61(0.46–5.68)	1.79(0.32–9.91)	1.93(0.33–11.16)
*p*-value	0.582	0.999	0.941	**0.045**	0.059	0.082	0.452	0.503	0.459
Intensive care^c^	0.93(0.17–5.07)	0.81(0.13–4.99)	0.89(0.14–5.75)	1.50(0.71–31.57)	1.22(0.56–26.74)	0.97(0.04–21.69)	0.78(0.18–3.38)	0.61(0.71–5.34)	0.68(0.07–6.29)
*p*-value	0.940	0.822	0.910	0.794	0.898	0.987	0.746	0.659	0.739
Medical Allied^d^	1.000	1.000	1.000	1.000	1.000	1.000	1.000	1.000	1.000

The reference category is Bangladesh/Afghanistan (combined n = 32). These two countries were combined together as reference category (Dependent variable) in regression analysis as the response distribution was less in this group as compared to the other three countries (independent variables)

^1^For specialty, Orthopedic Surgeons and General surgeons are group together as **Surgeons**

^2^General Physicians and Medical Intern are group together as **Physicians**

^3^Anesthesiologists, Pulmonary and Critical Care, and Infectious Disease Specialist are grouped together in to **Intensive Care**

^4^Rest of the specialties i.e. Cardiologist, Dermatologist, Otolaryngologists, Gynecologist/Obstetrician, Hematologist/Oncologist, Neurologist, Ophthalmologist, Pediatrician, Pathologist, Psychiatrist, and Radiologist are group into **Medical Allied**

**Model 1** = Crude Odds ratio (95% confidence interval) for univariate logistic regression

**Model 2** = Adjusted Odds ratio (95% confidence interval) for age in multivariate logistic regression

**Model 3** = Adjusted Odds ratio (95% confidence interval) for age and gender in multivariate logistic regression

## Discussion

Our study reported increasing numbers of deaths in physicians from COVID-19 in South Asia. This can be deduced by the increased mortality ratio of physicians in South Asian countries (7) in our study, in comparison to the mortality ratio of physicians from developed countries such as Italy (<1), Spain (<1), United States (<1), United Kingdom (<1), France (<1), and South Korea (<1), as reported by Yoshida et al [15].  The poor health care management systems, in comparison to developed countries like the USA [17], of some of the countries and extreme shortage and hoarding of PPEs in the region might be the major contributing factors that put physicians into a greater threat of death from the disease [17–19]. Common findings to all South Asian countries included a greater proportion of deaths of Internal Medicine specialists and male physicians. This could be because internal medicine specialists play a significant role at the frontline by offering patient-centered care [20] and male sex is associated with severe complications of COVID-19 respectively [21]. Increased age is one of the risk factors for the complications of COVID-19 [21]. The median age of physician mortality in our study was 60 years. Ing et al., also reported that 90% of the physicians dying from COVID-19 were males and two-third of them were over 60, however, physicians from all specialties were reported to be affected by the disease including dentists [11]. Kursumovic et al. also analyzed extensive data of Health Care Workers (HCWs) deaths due to COVID-19, which concluded that HCWs were at risk of contracting COVID‐19 through their occupational exposure [9]. Previously, epidemiological studies carried out in South Asia have linked communicable diseases with increased mortality in HCW’s [22].

Iran reported the highest proportion of physician deaths in our study and this has been previously reported by Ing et al. where they concluded Iran had the second-highest physician mortality in the world as of May 2020 [11]. In March 2020, Iran became one of the epicenters of infection [23] and there are many reasons why the country faces a relatively difficult situation [23–25]. Over the past 4 decades, Iran has tried to implement various methods to improve its health care system, however, many barriers have been preventing the actual implementations of these reforms [26]. In contrast, updated numbers of physician mortality in the USA, with the highest number of total cases in the world, was only 27 [24]. This highlights that a robust and competent healthcare management system could play a major role in mitigating the effects of communicable diseases on the front-line HCW’s.

Although much of the findings were similar amongst the major countries of South Asia, individual characteristics were worthy of mention. The mortality rate in India per 1000 in the general population was the lowest amongst all countries. Even though COVID-19 presents a tough battle due to the scarce healthcare resources in the country [27], the Indian government continues to assure that the provision of PPE is their priority. Moreover, ordinances against physician violence have been issued that commit to protecting HCWs [28, 29]. Afghanistan faces a rather arduous battle with COVID-19 where since the early 1970s, civil war has left a poor healthcare system in the country, and domestic conflicts and social taboos during the pandemic further complicate the situation [27]. This is further highlighted as in our study that the highest physician mortality rate per 1000 deaths in the general population was recorded from Afghanistan and still, concerns of low testing rate, inadequate reporting, and asymptomatic cases increase the suspicion of under-reporting of deaths [30].

The majority of the deaths of medical interns were reported from Pakistan with the median age of mortality being the lowest amongst all countries of the region. This could be mainly because young physicians like medical interns are perceived as first-line service providers in hospitals of the country [31]. Long shift hours during an intern year and vulnerability to the increased influx of infected patients could be a crucial factor attributed to the increased number of deaths in these individuals [32]. Pakistan also reported an increased number of physicians’ deaths who were affiliated with the public sector hospitals. Therefore, this may well suggest that Pakistan’s public health care system has not been equipped enough to meet the demands of the pandemic. Another important aspect in our results was the public health sector was significantly associated with mortality in multivariate analysis. There can be many factors related to this outcome, such as increased workloads, and exhaustion of personnel protection equipment (PPE) as our study reports the figures from the early days of the pandemic when the first wave of the virus spread was at its peak in Pakistan [33]

Our study highlights that some countries in South Asia reported deaths due to COVID-19 with a high mean physician mortality rate. One of the major contributory factors was the lack of PPE and the economic burden that confines nations from implementing ways to stop further physician deaths. Nevertheless, it could be very likely for these figures to increase as the second and third wave has already hit the majority of South Asia. Ways to prevent the misuse, hoarding, and black-marketing of PPEare strongly recommended. An overhaul of the entire healthcare infrastructure and management is needed in the region to tackle the current and any prospective epidemic or pandemic that may arise. It is crucial that international organizations such as World Health Organization, World Bank, United Nation Development Programme, Doctors without Borders, etc., and respective national policy-making organizations collectively collaborate to help alleviate the crisis that these countries of the region face.

Our study highlights the paucity of data regarding physicians’ mortality in healthcare emergencies such as the current pandemic as this directly contributes to the negligence regarding occupational health in South Asia. The importance of giving additional incentives to physicians is also evident from the study and better policies to protect them in every situation seem crucial. Collaboration among the South Asian countries could help policymakers and stakeholders to promote and build policies for primary healthcare [3]. Adopting the WHOs Health Worker Safety Charter can help protect physicians in the region to a great extent. The charter includes 5 steps that if incorporated into the policies could ensure the safety of HCWs. Proper reporting, educating, implementing strict laws, making helplines and guidelines available to physicians as well as ensuring that physicians are working under proper protection should be investigated [34]. Some inspiration can also be taken from UK’s NHS employers’ workplace health and safety standards. These mainly focus on the importance of competent leaders, teamwork, and efficient organization in healthcare settings for the policies to be implemented religiously [35].

Our study has some limitations. In our study, data on physician mortality numbers and characteristics were collected from newspaper websites and other online reporting sources. However, the veracity and adequacy of the collected data are restricted and insufficient in terms of political and government-based interests influencing and even leading to underreporting of the physicians’ mortality numbers. For example, although reported physician deaths from Sri Lanka, Nepal, and the Maldives were none, there is a possibility of physician deaths in these countries that were not reported by mainstream news media outlets. Although relying majorly on websites and social media resources might not be the most efficient way to analyze the physicians’ mortality, this might be the only available resource from the countries included in our study, especially the ones that influence reporting of news media outlets. Furthermore, the rapidly changing mortality-based statistics of the pandemic, low diagnostic testing and reporting rates, and unavailability of government-provided data from each country should be considered. Therefore, our study data were accurate as of August 31st, 2020. These limitations stress the need to take steps to formulate an independent international occupational medicine organization that aims to collecting authentic data about the physician's death toll in each country during the current and also future pandemics.

## Conclusion

Our study highlights how the COVID-19 pandemic is affecting the healthcare workforce in the region as we spotlight increased physician mortality numbers from South Asia. The reasons can be majorly attributed to decreased PPE, the highly infectious nature of the virus added to the poor health care management systems of the countries in the region to combat the pandemic. The highest number of physician deaths in the region was recorded in Iran whereas physician mortality characteristics show an increased number of deaths of internal medicine specialists and men. Future studies should be aimed to provide a further account of the detailed characteristics such as individual co-morbidities, socio-economic status, physician burnout, and main complications arising due to COVID-19 infection in physicians.

## Data Availability

Data sharing not applicable to this article as no data-sets were generated or analyzed during the current study.

## Abbreviations

MD: Doctor of medicine
SPSS: Statistical product and service solutions
USA: United States of America
STROBE: Strengthening the reporting of observational studies in epidemiology
COVID-19: Coronavirus Disease 2019
E.S: Emad Sajid
S.A: Saniya Ahmad
S.A.F: Syed Ali Farhan
N.N.J: Nadia Nazir Jatoi
HCWs: Health Care Workers
WHO: World Health Organization
UNDP: United Nation Development Programme
PPEs: Personal protective equipment
